# Ester-Producing Mechanism of Ethanol O-acyltransferase* EHT1* Gene in* Pichia pastoris* from Shanxi Aged Vinegar

**DOI:** 10.1155/2019/4862647

**Published:** 2019-01-03

**Authors:** Jia Chen, Ruipeng Nan, Rufu Wang, Lixin Zhang, Junfeng Shi

**Affiliations:** ^1^College of Horticultural Science, Shanxi Agricultural University, Taigu, Shanxi 030801, China; ^2^College of Animal Science and Veterinary Medicine, Shanxi Agricultural University, Taigu, Shanxi 030801, China; ^3^College of Food Science and Engineering, Shanxi Agricultural University, Taigu, Shanxi 030801, China; ^4^Institute of Farm Product Storage and Fresh-Keeping, Shanxi Academy of Agriculture Science, Taiyuan, Shanxi 030031, China

## Abstract

The ethanol O-acyltransferase* EHT1 *is an important element of key signaling pathways and is widely expressed in yeast strains. In this study, we investigated the expression of* EHT*1 in the overexpression lines or knockout system of* Pichia pastoris* using qRT-PCR and western blotting. The amount of total protein was determined using the Bradford method; the esterase activity was determined using p-nitrophenyl acetate as a substrate, and the production of volatile fatty acids in wild-type, knockout, and over-expression systems was detected using SPME GC-MS. The esterase activity of* EHT*1-knockout* P. pastoris* was significantly lower than that in wild type (*P*<0.01), and the activities of esterase in three* EHT*1-overexpressing strains—OE-1, OE-2, and OE-3—were significantly higher than those in wild type (*P*<0.01). In the* EHT*1-knockout strain products, the contents of nine volatile fatty acids were significantly lower than those in wild type (*P*<0.01), and the relative percentages of three fatty acids, methyl nonanoate, methyl decanoate, and ethyl caprate, were significantly lower than those in the other six species in the wild-type and knockout groups (*P*<0.05). The nine volatile fatty acids in the fermentation products of the overexpressed* EHT*1 gene were significantly higher than those in the wild-type group (*P*<0.01). The relative percentages of the three fatty acid esters, methyl nonanoate, methyl caprate, and ethyl caprate, were significantly higher than those in the other six species (*P*<0.05).* EHT*1 plays an important regulatory role in esterase activity and the production of medium-chain volatile fatty acids.

## 1. Introduction

Shanxi aged vinegar is fermented through traditional solid-state fermentation using Shanxi's unique Daqu as a starter. Its final physicochemical properties and volatile flavor are influenced by the yeast [1, 2].* Saccharomyces cerevisiae* cells produce a substantial amount of esters during fermentation, significantly affecting the complex flavor of foods and fermented alcoholic beverages [3]. Detection of volatile aroma components in the yeast strains isolated from Shanxi aged vinegar yielded various ester species, such as ethyl caprylate and ethyl hexanoate [4]. The studies have shown that the ethyl hexanoate in yeast is synthesized from hexanoyl-CoA and ethanol by an alcohol caproyl transferase-catalyzed reaction or from hexanoic acid and ethanol under the catalysis of esterases [3, 5–7]. Yeast ethyl ester synthesis is catalyzed by ethanol O-acyltransferase and is encoded mainly by* EHT*1 and* EEB*1, which belong to the ternary gene family (*EHT*1,* EEB*1, and* YMR210w*) [8, 9].

**Figure 1:**
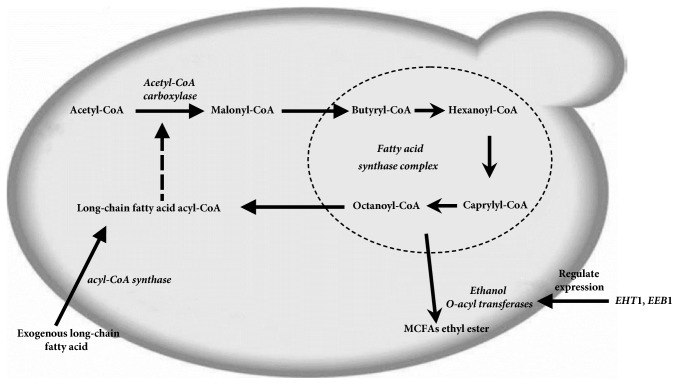
Fatty acid biosynthesis and its relationship with medium-chain fatty acid ester formation in* Pichia pastoris*. Dashed ellipse represents fatty acid synthase complex; dashed arrow represents the inhibition of long chain fatty acid acyl-CoA on acetyl-CoA carboxylase.* EHT*1 and* EEB*1 genes regulate the expression of ethanol O-acyltransferase.

The rate of ethyl hexanoate formation depends mainly on the substrate concentrations (hexanoyl-CoA and ethanol) and enzymatic activity [10]. Some scholars have proposed alcohol acyltransferase (named Eht1 (ethanol hexanoyl transferase I)) as a candidate for ethyl ester synthase [3]. The* EHT*1 gene product (Eht1) in wine yeast plays an important role in the production of ethyl ester (ethyl hexanoate, ethyl octanoate, and ethyl decanoate) in medium-chain fatty acids (MCFA) [11, 12]. The results of the purification of GST–Eht1 and GST–Eeb1 fusion proteins clearly showed that these proteins regulate the enzymatic activities of both MCFA ethyl ester synthesis and hydrolysis. The absence of* EHT*1 or* EEB*1, especially the deletion of both, led to a significant reduction in the production of most MCFA ethyl esters (Figure 1). The Eht1 enzyme has the largest contribution to the production of MCFA ethyl esters [8]. We found that yeasts affected the final chemical and volatile flavor composition of Shanxi aged vinegar.* Pichia pastoris* is the main ester-producing yeast and has an important contribution to flavor.* Pichia pastoris* can tolerate the extreme production conditions of low pH, low water activity, high osmotic pressure, and anaerobic conditions of Shanxi aged vinegar. The oxygen-limited conditions can induce* P. pastoris* to carry out alcoholic fermentation, activate key enzymes in the fermentation pathway, and contribute to the increase of metabolites such as ethanol, glycerol, and ethyl acetate.

Analysis of enzymes controlling ester synthesis in yeast established that* EHT*1 and* EEB*1 mainly control short- and medium-chain fatty acid esters. After studying the distribution of* EHT*1 and* EEB*1, it was found that the* EHT*1 exists in all strains. It is thus speculated that the* EHT*1 gene is essential for ester synthesis in yeast. Hence, in this study, endogenous* EHT*1 was overexpressed or knocked out in* P. pastoris*. Further understanding the role and mechanisms of* EHT*1 in yeast will allow for more control of the fermentation process in a variety of foods. Yeast strains may be tailored to produce esters of interest at desired quantities, resulting in unique flavor profiles.

## 2. Materials and Methods

### 2.1. Microbes and Culture Conditions

*Escherichia coli *DH5*α* was used for cloning and vector maintenance. The host* P. pastoris *strain GS115 and pPIC9K plasmid were from Invitrogen (Carlsbad, CA, USA). DH5*α* was grown in LB medium at 37°C.* P. pastoris *was cultured in YPD medium (1 % yeast extract, 2 % peptone, 2 % dextrose) and BMGY medium (1 % glycerol, 1 % YE, 2 % peptone, 1.34 % yeast nitrogen base w/o amino acids and ammonium sulfate (YNB), 0.0004% biotin at pH 6.0, and 100 mM potassium phosphate buffer) or BMMY induction medium (methanol instead of glycerol). The growth conditions for* P. pastoris *were 28–30°C and 300 rpm shaking.

### 2.2. *EHT*1 Cloning and Transformation

According to the* P. pastoris* sequence of* EHT1* in GenBank (Accession no. FR839628),* EHT*1_*EcoR*I_F (5′-TTTGAATTCATGCCATCTTGGGGGTTCC-3′) and* EHT*1_NotI_R (5′-ATAAGAATGCGGCCGCAATTCCAGTTTTATAGCTG-3′) primers were designed.* EcoR*I and* Not*I enzyme were added to forward and backward primers, respectively. The synthetic* EHT1 *gene was cloned into the* EcoR*I/*Not*I cleavage sites of the expression vector pPIC9K, under the control of a methanol-inducible (AOX1) promoter and directly downstream of an *α*-factor secretion signal sequence. The recombinant expression plasmid was linearized by digestion with* Sal*I restriction enzyme and transformed into* P. pastoris *GS115 by electroporation, using the internal preset protocol (BIO-RAD Gene Pulser Xcell Electroporation System, Hercules, CA, USA). After the transformation, the cuvette contents were spread on MD medium (1.34 % YNB, 0.00004 % biotin, 2 % dextrose) and incubated at 30°C for 5 days. The clones that appeared on the MD medium were restreaked step-wise on YPD medium containing 0.2 mg/mL, 0.5 mg/mL, 1 mg/mL, or 2 mg/mL G418 for selection of* EHT*1 high-expression strains.

### 2.3. *EHT*1 Gene Knockout and Transformation

We used homologous recombination methods to knock out the* EHT*1 gene. The gene KO was performed using* Agrobacterium tumefaciens*-mediated transformation (ATMT) methods [13]. The forward and reverse sequences of* P. pastoris* GS115* EHT*1 were retrieved from GenBank. The primers* EHT*1O1(5′-GGTCTTAAUTGGAAAATACCTGGGCCAGT-3′) and* EHT*1O2 (5′-GGCATTAAUCTGTCAAACGAACCTGCACA-3′) were used to amplify the left arm of the* EHT*1 gene. The primers* EHT*1A3 (5′-GGACTTAAUGTTGGCGCCCCTATTGATTT-3′) and* EHT*1A4 (5′-GGGTTTAAUCGGTGGCAAAGTGAAAGTGA-3′) were used to amplify the right arm of the* EHT1* gene. PCR fragments were ligated in the vector pRF-HU2 [14] and transformed into DH5*α* cells. Both recombinant arms in one clone from PCR amplification were sent for sequencing to validate the presence of the inserts in* E. coli* transformants. The correct clone was propagated in LB medium, and the plasmids were extracted using the Easy Pure Plasmid MiniPrep Kit (cat. no. EM101-01, TransGen Biotech Co., Ltd., Beijing, China) according to the manufacturer's manual.

MiniPrep plasmid was transformed into* A. tumefaciens* using a standard electric shock method.* A. tumefaciens* containing plasmid was resuspended, added to a flask with LB medium containing 20 *μ*g/mL rifampicin, 25 *μ*g/mL kanamycin, and 75 *μ*g/mL carbenicillin, and cultured until the OD reached 3-4. YPD liquid medium was inoculated with one clone from solid YPD medium and cultured until the OD reached 1-2. Both microbes were collected, resuspended, and washed three times in IMAS liquid medium [14].* A. tumefaciens* and* P. pastoris* were cocultured in IMAS solid medium containing 20 *μ*g/mL rifampicin, 25 *μ*g/mL kanamycin, and 75 *μ*g/mL carbenicillin for 3-4 days until clones appeared.

### 2.4. *EHT*1 Gene Mutant Identification

Selected colonies of transgenic yeast were subjected to colony PCR to directly test for* P. pastoris *mutant* EHT*1 clones, and wild-type* P. pastoris* was used as the control. The cells were lysed by three rounds of heat and liquid nitrogen treatment. After centrifuging, the genomic DNA (gDNA) was used as a template for PCR, using primers specific to the* Hyg* gene, the selection marker (forward: 5′-AGCTGCGCCGATGGTTTCTACAA-3′ and reverse: 5′-CGCGTCTGCTGCTCCATACAA-3′). The standard cycling conditions used for PCR were as follows: 3 min at 95°C, 30 cycles of 20 s at 95°C, 20 s at 58°C, 45 s at 72°C, and one cycle of 5 min at 72°C. EHT1O1(5′-GGTCTTAAUTGGAAAATACCTGGGCCAGT-3′) and RF-2 (5′-TCTCCTTGCATGCACCATTCCTTG-3′) were used to test for correct crossover at the left flank.* EHT*1A4(5′-GGGTTTAAUCGGTGGCAAAGTGAAAGTGA-3′) and RF-1 (5′-AAATTTTGTGCTCACCGCCTGGAC-3′) were used to test for correct crossover at the right flank. RF-1 and RF-2 were located on the vector, and there was a corresponding band in both PCR amplifications compared with the WT* P. pastoris*.

### 2.5. Extraction of RNA and Reverse Transcription to cDNA

Total RNAs from* P. pastoris* were extracted from WT, KO, and OE strains cultured on YPD solid medium for 48 h. The strains were scraped from the solid medium, ground with quartz sand under liquid nitrogen protection, and then processed using TRIzol reagent (Invitrogen, Carlsbad, CA, USA). The RNA was reverse-transcribed to cDNA with random primers, and conditions recommended by the provider were followed (TransGen Biotech Co., Ltd., Beijing China).

### 2.6. Real-Time PCR Quantification of* EHT*1 in Overexpression and Mutant Lines

Real-time PCR was used to quantify the* EHT*1 expression level in OE and mutant lines. Primers for* EHT*1 gene were designed (qRT-*EHT*1F, 5′-AGTGGGATGGTGGATTCTGG-3′ and qRT-*EHT*1R, 5′-TGTGCAGGTTCGTTTGACAG-3′).* P. pastoris* actin (Accession no. CAY68002.1) was used as a reference gene (qRT-ActinF, 5′-GTTTGCGCTGCTATGAATGC-3′ and qRT-ActinR, 5′-GAGCAACATCCCTGATTCCG-3′).

The real-time PCR kit was used according to the manufacturer's instructions (TransGen Biotech Co., Ltd., Beijing, China). The PCR reactions were performed in a Roche real-time PCR System (Roche Diagnostics Ltd., Basel, Switzerland), and the conditions were based on manufacturer recommendations. Melting curve analyses of amplification products were performed at the end of each PCR to ensure that unique products were amplified. The data were processed using the 2^−ΔΔCt^ method.

### 2.7. Eht1 Expression in* P. pastoris*

Five mL of BMGY medium was inoculated with one colony of WT, KO, and OE strains and incubated at 30°C until an OD_600_ of 2-4 was reached. The cells were collected by centrifugation, washed three times with sterilized water, and then induced by methanol in BMMY induction medium at 30°C. Methanol was added to the flasks every 24 h to maintain the final methanol concentration at 1 %. The culture supernatants were harvested by centrifugation, and the expressed recombinant Eht1 was analyzed by sodium dodecyl sulfate-polyacrylamide gel electrophoresis (SDS-PAGE).

### 2.8. Eht1 Western Blotting Analysis

SDS-PAGE was used to determine the purity and molecular mass of the enzyme using a 5% (w/v) stacking gel and a 10% (w/v) separating gel. The samples from broth were boiled 10 min with protein loading buffer for SDS-PAGE analysis. The bands were transferred to a nitrocellulose membrane (NC film) with a semidry transfer instrument (15 V, 30 min). The NC membrane was blocked by BSA (50 g/L, in blocking solution) overnight in 4°C and washed five times (1 × PBST containing 0.5 mL/L Tween-20, 3 min each time). EHT1 rabbit antibody (Hefei Zhien Biology Co., Ltd., Hefei, China) was diluted 1: 2,000 in blocking solution. The NC film was incubated 1 h with primary antibody and then washed five times with 1 × PBST as above. After washing, the NC film was dipped in HRP-labeled goat anti-rabbit secondary antibody, which was diluted 1:5,000 in blocking solution, for 1 h at room temperature. At last, the NC was washed five times for exposure. The antibody (cat. no. 1109, Ayabio Biological Technology Co., Ltd., Chongqing, China) for* P. pastoris β*-actin (ID: P60709, Swiss-Prot) was used as a reference load and processed following the manual.

### 2.9. Measurement of Protein Concentration and Esterase Activity

The amount of total protein was determined by the Bradford method, using bovine serum albumin (BSA) as a standard. Esterase activity was measured as described previously [15]. Briefly, a stock solution of 100 mmol/L pNPA (p-nitrophenyl acetate, Sigma-Aldrich Co. LLC., St. Louis, MO, USA) was prepared in CH_2_Cl_2_ as substrate. Before initiation of the assay, 40 uL of the stock solution was added to 40 mL of buffer solution (20 mmol/L Tris-HCl, pH 8.0, 150 mmol/L NaCl, and 0.01 % Triton-X-100). Two hundred *μ*L of crude extract was then incubated with 2 mL of substrate solution in test tubes at 30°C for 30 min. After incubation, the absorbance was measured at 405 nm in an ultraviolet-visible spectrophotometer against a blank without the protein. One unit of enzyme activity was defined as the amount of the enzyme releasing 1 *μ*mol of pNPA per min at 30°C, pH 8.0. Samples obtained from WT* P. pastoris* GS115 were tested as a control. The experiment was conducted in triplicate.

### 2.10. SPME GC-MS Analysis

SPME GC-MS analysis was performed as previously described [16]. Briefly, each sample (8 mL) was placed in a 15-mL vial and analyzed using SPME (solid-phase microextraction) coupled with GC-MS (gas chromatography-mass spectrometry). A 100-*μ*m polydimethylsiloxane coating fiber (Sigma-Aldrich Co. LLC., Shanghai, China) and a manual SPME holder (Sigma-Aldrich Co. LLC., Shanghai, China) were used after preconditioning according to the manufacturer's instructions. The samples were equilibrated at 45°C for 30 min, and subsequently the SPME fiber was introduced into the vial headspace. After 30 min, the fiber was removed from the vial and immediately inserted into the GC injection port for a 5-min sample desorption. GC-MS was carried out using a Bruker SCION SQ GC-MS (Billerica, MA, USA) equipped with a DB-5 capillary column. Injector, detector, and ion source temperatures were 250, 280, and 230°C, respectively. The conditions for GC analysis were as follows: The column is TR-5MS (30 m × 0.25 mm, 0.25 *μ*m). The inlet temperature was 300°C, the carrier gas He, and the flow rate was 2 mL/min. The injection volume is 350 *μ*L and the split ratio is 20:1. Heating program was as follows: starting temperature 40°C, hold for 3 min, heat up to 180°C at 3°C/min for 5 min, and heat up to 250°C at 20°C/min for 5 min. MS conditions were as follows: electron bombardment (EI) ion source, electron energy 70 eV, ion source temperature 250°C, transmission line temperature 280°C, quadrupled temperature 180°C, and mass scan range m/z: 35-500. The results were analyzed using NIST software.

### 2.11. Statistical Analysis

All data were analyzed using the SPSS 17.0 software (SPSS Inc., Chicago, IL, USA). Statistical analysis was based on analysis of variance (ANOVA), and we also used the Duncan method for multiple comparisons. The data generated were converted to graphs using SigmaPlot 12.5 (Systat Software Inc., London, UK). Data represent the mean from triplicate experiments ± standard deviation (S.D.).* P*<0.05 was considered statistically significant.

## 3. Results

### 3.1. *EHT*1 Gene Cloning and Plasmid Construction

**Figure 2:**
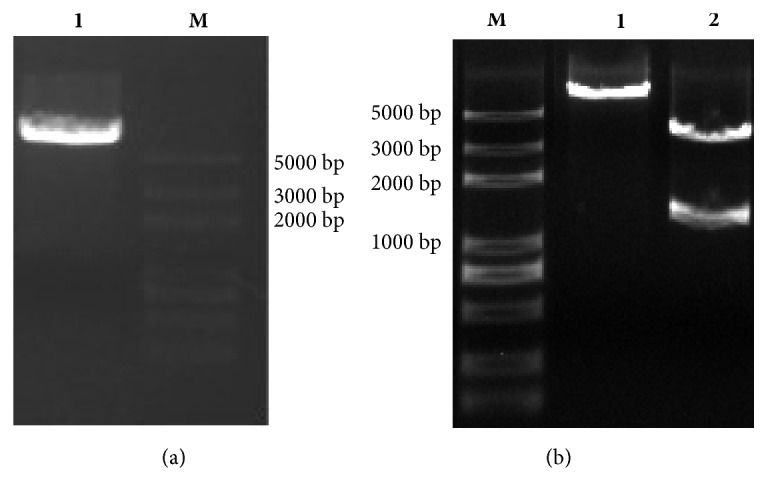
*EcoR*I + *Not*I double-enzyme-cut vector to identify its correction for *EHT*1 gene. (a) Line I: pPIC9K; Line M: marker; (b) Line M: marker; Line 1: pPIC9K with *EHT*1; Line 2: Line 1 cut by *EcoR*I and *Not*I.

pPIC9K-*EHT*1 agarose electrophoresis bands of pPIC9K-*EHT*1,* EcoR*I, and* Not*I double-enzyme cut were in line with expectations: they were single bands of high intensity and met the test requirements; they could be used for subsequent plasmid extraction and mRNA level testing (Figure 2).

### 3.2. *EHT*1 Gene Knockout Identification on DNA Level

**Figure 3:**
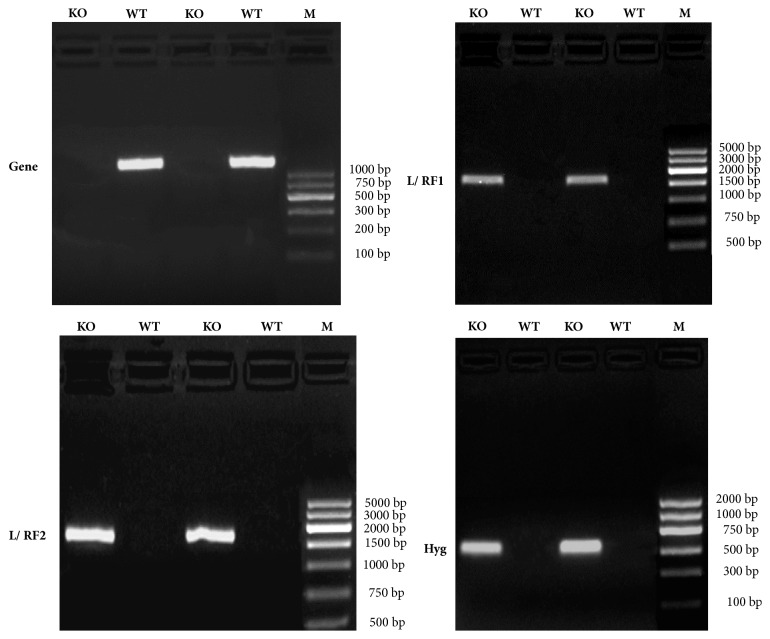
Identification of KO on DNA level. gDNA from KO and WT strains was used as the template. Primers for* EHT*1 gene, recombinant arm (left L/RF1 and right R/RF2), and selection marker (*Hyg*) amplified relevant sequences. In KO mutant, L/RF1, R/RF2, and* Hyg* should produce bands indicative of the insertion of the vector, while WT should lack vector sequence bands.* EHT*1 gene was not present in KO mutant compared with WT.

KO and WT gDNA were used as templates. The* Hyg* gene was selected as an internal control, and the recombinant arms (the L/RF1 and R/RF2 sequences) were amplified. From Figure 3, we can see that the band of interest is single, while RF-1 and RF-2 are on the vector; there is no band of PCR amplification from KO as compared with WT* P. pastoris*, which shows that* P. pastoris EHT1*KO strains were constructed successfully.

### 3.3. qRT-PCR for* EHT*1 KO and OE

**Figure 4:**
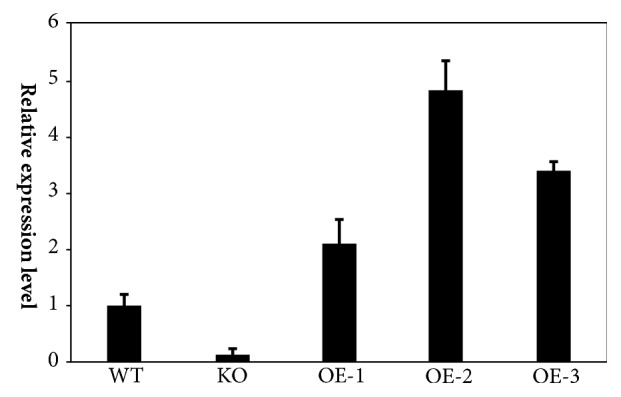
qRT-PCR for quantification of the expression level of* EHT*1 in KO and OE. WT was used as the reference control. Three lines of OE (OE-1, OE-2, OE-3), one line of KO, and one line of WT were tested. Each line represents triplication.* P. pastoris* actin was used as the reference gene.

The mRNA expression levels of* EHT*1 in KO and OE strains were quantified using qRT-PCR. The test results showed that the expression level in KO was significantly lower than that in OE and WT (*P*<0.01). The mRNA expression levels in OE-1, OE-2, and OE-3 were significantly higher than those in the WT group (*P*<0.01), while the mRNA expression level in OE-2 was significantly higher than that in the OE-1 and OE-3 groups (*P*<0.05) (Figure 4).* P. *pastoris recombinant OE strains could be selected from the OE-2 group, which indicated that* EHT*1 KO strains were successfully constructed in terms of mRNA expression levels.

### 3.4. Eht1 Expression in* P. pastoris*

**Figure 5:**
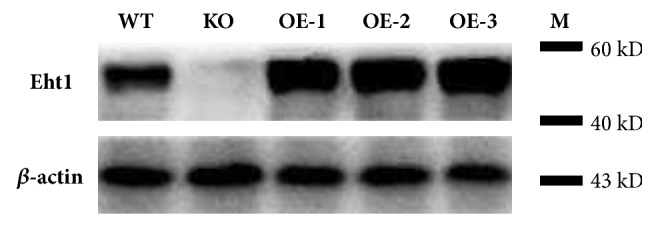
Western blotting analysis of Eht1 expression in KO, WT, and OE groups. Please note that the Eht1 proteins are all intracellular. *β*-actin was used as a loading control. The results show that there was Eht1 protein accumulation in* P. pastoris* cells of the OE groups, and those of the KO group did not express Eht1.

**Figure 6:**
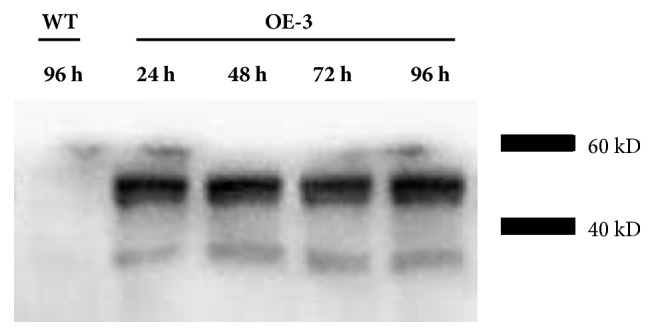
Time course of the extracellular expression of Eht1 in WT and OE. Samples were analyzed 24 h, 48 h, 72 h, and 96 h after OE induction. The cultures were adjusted to 2 OD, and 1 mL was centrifuged at 10,000* g* for 15 min at 4°C. The supernatants were collected, and 20 *μ*L was separated by SDS-PAGE. WT at 96 h was used as the control.

The Eht1 expression levels in KO, WT, and OE were analyzed by western blotting. There was a large amount of Eht1 protein accumulated in* P. pastoris* OE-1, OE-2, and OE-3 groups and no Eht1 expression in KO (Figure 5). The total protein in the* P. pastoris* OE groups was extracted after 24 h, 48 h, 72 h, and 96 h of culture. The WT group at 96 h was used as a control for western blotting. The expression of Eht1 protein in* P. pastoris* OE groups was significantly higher than that in the WT group at 24 h, 48 h, 72 h, and 96 h (*P*<0.01), which indicated that the KO and OE strains were successfully constructed in terms of Eht1 protein level. The endogenous* EHT*1 gene in* Pichia* is of great significance (Figure 6).

### 3.5. Measurement of Esterase Activity

**Figure 7:**
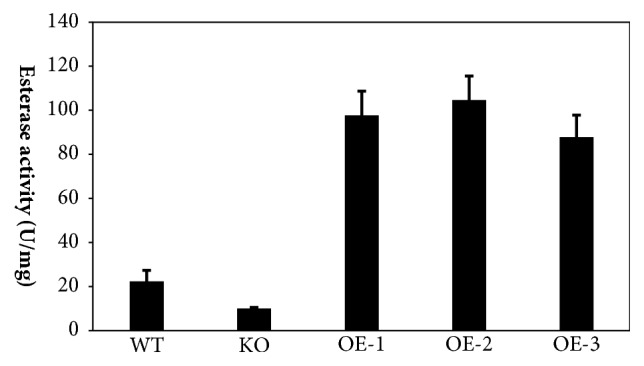
Esterase activity was determined using p-nitrophenyl acetate as a substrate. The total protein amount was determined by the Bradford method, using bovine serum albumin (BSA) as a standard. Three lines of OE (OE-1, OE-2, OE-3), one line of KO, and one line of WT were tested. Each line represents triplication. The samples were collected after 72 h of methanol induction.

Figure 7 shows the esterase activity determined using p-nitrophenyl acetate as a substrate. Based on the results, the esterase activity of* P. pastoris EHT*1 KO was significantly lower than that of the control group (*P*<0.01). The enzymatic activities of the three* EHT*1 OE strains of* P. pastoris*, OE-1, OE-2, and OE-3, were significantly greater than that of the WT group (*P*<0.01). The esterase activity in the OE-2 group was significantly higher than that in OE-1 and OE-3 groups (*P*<0.01). These results indicated that the OE-2 strain was successfully constructed, which further demonstrated the important regulatory role of the* EHT*1 gene in the esterase activity in* P. pastoris* fermentation products.

### 3.6. SPME GC-MS Analysis

**Figure 8:**
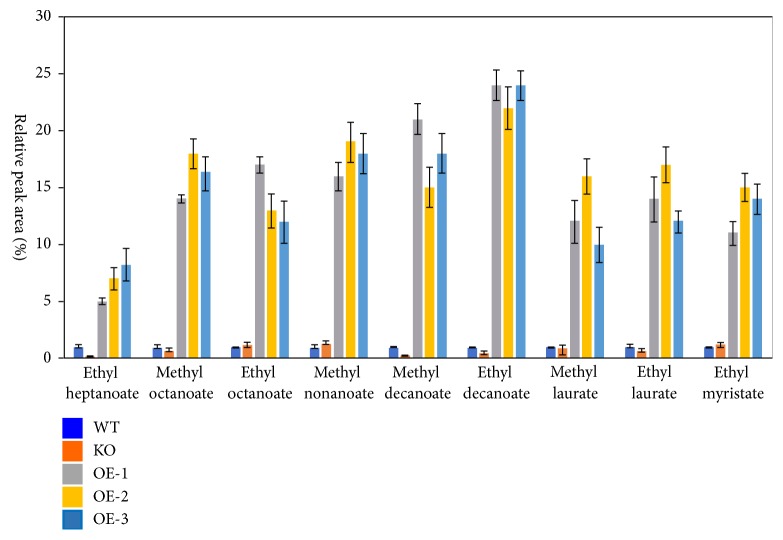
Production of volatile compounds in WT, KO, and three lines of OE measured by SPME GC-MS. Strains were allowed to ferment for 24 h at 30°C, and volatile compound production was subsequently induced with 0.5 % methanol for 48 h. Amounts of individual volatile compound are shown as percentage of total aromas. Data represent the mean from triplicate experiments ± standard deviation.

Volatile fatty acid production in WT, KO, and three species of OE was examined using SPME GC-MS (Figure 8). As shown in Figure 8, after the* EHT*1 gene KO, the ethyl caprylate, methyl caprylate, ethyl caprylate, methyl pelargonate, methyl caprate, ethyl decanoate, laurate ester, ethyl laurate, and ethyl myristate levels in the* P. pastoris* fermentation products were significantly lower than those in the control group (*P*<0.01). The relative percentages of the three fatty acids methyl nonanoate, methyl decanoate, and ethyl caprate were significantly lower than those of the control group and percentages of ethyl heptanoate, methyl octanoate, ethyl octanoate, methyl laurate, ethyl laurate, and ethyl myristate in the KO group (*P*<0.05). This indicated that* P. pastoris *esterase* EHT*1 exerts greater control of the synthesis of short-chain fatty acid esters.

In the OE-1, OE-2, and OE-3* P. pastoris* strains with overexpressed* EHT*1 gene, the relative percentages of ethyl heptanoate, methyl octanoate, ethyl octanoate, methyl nonanoate, methyl caprate, ethyl caprate, methyl laurate, ethyl laurate, and ethyl myristate in the fermentation products were significantly higher than those in the WT group (*P*<0.01); the relative contents of the three fatty acids methyl nonanoate, methyl caprate, and ethyl caprate were significantly higher than those of ethyl heptanoate, methyl octanoate, ethyl octanoate, methyl laurate, ethyl laurate, and ethyl myristate (*P*<0.05). This further indicated that the* EHT*1 gene in* P. pastoris *exerts greater control of the synthesis of short-chain fatty acid esters. The results showed that the* EHT*1 gene plays an important regulatory role in the production of volatile fatty acids in the fermentation products of* P. pastoris*.

## 4. Discussion

Fatty acid ethyl esters are secondary metabolites produced during microbial fermentation. Volatile MCFA ethyl esters are important flavor compounds and major sources of the fruity aroma in beer, wine, and vinegar ([10], Robinson et al., 2014). The biosynthesis of fatty acid ethyl esters in yeast requires an enzymatic reaction by ethanol O-acyltransferases (AEATases), which catalyze the conversion of fatty acyl groups from acyl-CoA to ethanol [3, 8, 15]. MCFA ethyl esters, including ethyl hexanoate, ethyl octanoate, and ethyl caprate, are produced in the aromas of apple, fennel, and flowers, respectively. In this study, the production of volatile fatty acids in WT, KO, and three OE species was detected using SPME GC-MS. After* EHT*1 gene KO, the levels of ethyl caprylate, methyl caprylate, ethyl caprylate, pelargonic acid, methyl caprate, ethyl caprate, methyl laurate, ethyl laurate, and ethyl myristate in the* P. pastoris* fermentation products were significantly lower than those in the control group. The relative percentages of three fatty acids, methyl pelargonate, methyl decanoate, and ethyl decanoate, were significantly lower than those in the control group and the percentages of ethyl heptanoate, methyl octanoate, ethyl octanoate, methyl laurate, ethyl laurate, and ethyl myristate in the KO group. This indicated that esterase* EHT*1 plays a more significant role in controlling the synthesis of short-chain fatty acid esters. This study further confirmed that the* EHT*1 gene producing ethanol O-acyltransferase has a similar function in the fermentation process of Shanxi vinegar.

*S. cerevisiae* may contain MCFA ethyl ester synthases other than Eht1 and Eeb1, as the production of MCFA ethyl ester under the double deletion of* EHT*1 and* EEB*1 in strains showed that* EHT*1 and* EEB*1 are responsible for the synthesis of most of the MCFA ethyl esters in yeast [8]. Although* EHT*1 and* EEB*1 double deletion led to a significant decrease in all MCFA ethyl ester production, MCFA production was the only element reduced, with the production of ethyl octanoate reduced by 70% and that of ethyl butyrate and ethyl caprate reduced by 50% [8]. This study found that, after* EHT*1*-*KO, the relative contents of methyl pelargonate and methyl caprate were significantly lower than those of the other fatty acids in the control group and KO group. It was thus speculated that the unique aroma of Shanxi aged vinegar, which is different from the aroma of liquor and wine, arises from methyl ester fatty acids. Hence, the* EHT*1 gene mainly regulates the production of methyl ester MCFA in the fermentation products of Shanxi aged vinegar.

Previous studies have confirmed that* ATF*1,* Lg-ATF*1, and* ATF*2 genes encode acetate synthase. Nevertheless, the double deletion of* ATF*1 and* ATF*2 does not affect the synthesis of MCFA ethyl esters [17–19], which suggests the presence of unknown genes involved in ester biosynthesis in yeast. It has been demonstrated in wine fermentation tests that* EHT*1-KO strains are viable [20] with a prolonged lag in culture growth [8]. The localization and posttranslational processing of the ester-forming gene is a major obstacle in the expression of eukaryotic membrane proteins in the prokaryotic system [21]. In this experiment, the mRNA expression levels of KO and OE of the* EHT*1 gene were quantified by qRT-PCR. The mRNA expression level of the KO group was significantly lower than that of the OE and WT groups (*P *<0.01), while the mRNA and protein expression of the OE-2 group was significantly higher than that of the OE-1 and OE-3 groups (*P*<0.05). The esterase activity after* P. pastoris EHT*1 gene KO was significantly lower than that of the control group (*P*<0.01), while the esterase activity of the three* P. pastoris* strains with overexpressed* EHT*1 gene was significantly higher than that of the WT group (*P*<0.01). This indicated the successful construction of the* EHT*1-overexpressing OE-2 strain, which further demonstrated the important role of the* EHT*1 gene in regulating the esterase activity of* P. pastoris* fermentation products.

## 5. Conclusions

The esterase activity after* P. pastoris EHT*1 gene KO was significantly lower than that of WT (*P*<0.01). In contrast, the esterase activities in the three* P. pastoris *strains with overexpressed* EHT1 *were significantly higher than that of WT (*P*<0.01). After* EHT*1 gene KO, the percentage of volatile fatty acids in the* P. pastoris* fermentation products was significantly higher than in the WT group (*P*<0.01). The contents of three fatty acids, including methyl nonanoate, methyl decanoate, and ethyl caprate, were significantly higher than those of the other fatty acids (*P*<0.05). The results indicated that* EHT*1-KO and -OE strains were successfully constructed in terms of the DNA, mRNA, and protein levels. The* EHT*1 gene plays an important role in regulating the esterase activity in* P. pastoris *and the volatile fatty acid production in fermentation products.

## Data Availability

The data used to support the findings of this study are available from the corresponding author upon request.

## Supplementary Materials

Supplementary Materials The* P. pastoris* sequence information of* EHT*1 in GenBank (Accession no. FR839628) and design site information of* EHT*1* EcoR*I F and* EHT*1* Not*I R primers for* EHT*1 cloning and transformation (Supplementary Material 1, 2 and 3). We provided the amplification system and amplification conditions required for PCR experiments. We used homologous recombination methods to knock out* EHT*1 gene. The gene was knocked out using Agrobacterium tumefaciens-mediated transformation (ATMT) methods. The primers* EHT*1A3 and* EHT*1A4 amplified the right arm of* EHT*1 gene (Supplementary Material 4: amplification the left and right recombinant arms). The up and down sequences of* P. pastoris EHT*1 were retrieved from the GenBank. The primers* EHT*1O1 and* EHT*1O2 were used to amplify left arm of EHT1 gene (Supplementary Material 5: the four primers below should be synthesized). We also provided the running PCR on genomic DNA to amplify recombinant region and ligation of the PCR products with the vector (Supplementary Material 6 and 7). It also provides information about the transformation of* E. coli* with the construct and provides the method of validating the presence of the inserts in* E. coli *transformants (Supplementary Material 8 and 9).
