# Supplementary material for: Ester-Producing Mechanism of Ethanol O-acyltransferase EHT1 Gene in Pichia pastoris from Shanxi Aged Vinegar
Source: Biomed Res Int. 2019 Jan 3;2019:4862647. doi: 10.1155/2019/4862647 (PMC6335666; doi:10.1155/2019/4862647)
Supplement: Supplementary Materials — The P. pastoris sequence information of EHT1 in GenBank (Accession no. FR839628) and design site information of EHT1 EcoRI F and EHT1 NotI R primers for EHT1 cloning and transformation (Supplementary Material 1, 2 and 3). We provided the amplification system and amplification conditions required for PCR experiments. We used homologous recombination methods to knock out EHT1 gene. The gene was knocked out using Agrobacterium tumefaciens-mediated transformation (ATMT) methods. The primers EHT1A3 and EHT1A4 amplified the right arm of EHT1 gene (Supplementary Material 4: amplification the left and right recombinant arms). The up and down sequences of P. pastoris EHT1 were retrieved from the GenBank. The primers EHT1O1 and EHT1O2 were used to amplify left arm of EHT1 gene (Supplementary Material 5: the four primers below should be synthesized). We also provided the running PCR on genomic DNA to amplify recombinant region and ligation of the PCR products with the vector (Supplementary Material 6 and 7). It also provides information about the transformation of E. coli with the construct and provides the method of validating the presence of the inserts in E. coli transformants (Supplementary Material 8 and 9). [file 4862647.f1.pdf]

According to https://www.ncbi.nlm.nih.gov/nuccore/328349772?from=1335369&to=1336727&sat=2&sat_key=46463764

EHT1 mRNA: Available without EcorI and NotI sites, available

ATGCCATCTTGGGGGTTCCATAATCAGATTCATGCTACCTACAGCGATAAATCGGTG
GCACTCAAGCTGAAGTCTGGCAAGAACGACTTTGCACCTTTGAATGTCATTCCTTTT
TCTACATTTATTAAGACTTTAGTACCTGAACTCAGCGATGGCTACAAATTCAACCTT
CACCCTTTTCTGTTCAATGGCGCTCTCCAGACTTTATACGCGTCCAAAGCTGACTAT
AGTAAAGAATTTCAAATATACTATGGAAGATGCATGGTTCGATATCCTGAGGCAAAT
CATGAGTATCCACATTTACATCTTGGTCAAGCGACTGCGGATTTTGTAGTTGATCCA
CCAACAGAAATCGATGAATGGAATTATTTATACAATCAGACTTTACCTGAAGGATGG
CCTCGATTGCATCCGCGGACTAGATTCCTAACAGTGCAAGAAGAACAAGACCTGGAA
GCTGACTGGGCCTCAAACTCTGGATCAATTGTTGTAATTATTCACGGATTGGCCGGT
GGCTCGCACGAACCGGGAATACGAGATGTGAGCCAGCATCTTCATCAAGCTGGTTTT
AATGTTGTGACACTCAACTCTCGTGGATGTTGTCGCTCTAAATTGTCAACAGGAAGA
CTTTACTCGGCCGTCGAAACAGATGATCTCCGTTACTTTATTGATGAGCTACATAAG
AAAATACCCAACAAGCCTATCTATCTTTTGGGATTTTCTTTAGGGTCTGCACTGGTT
CTCAATTATCTTGGCGAGGAAGGAAAGAAGAGCTTCATCAAAAGTGCGGTCACTATC
GGAGCACCTGTAGACCTCCTGGACTCCCATTATCATTTGAACCACAGTTACTCTGGA
AAGTATCTATTCGATCCTGCAGTGGCTTCATTCTTGTCAAATTTGGTAAAAGTTAAC
TTCCCAATTCTAAACCGTGATAAACCGGAAGTATTCTCATACGAAAAGAAGTCCAAA
GATTACTTGGTACGGAGAATCGTCGACTTTGATCGAAAATATACTGCCCCAGCGTAT
GGATTTGCAAGTCCAAAATATTACTATCAAGCTGGATCTCCCTTAACCAAGCTAGTA
AATATTTACACTCCAACAGTACTGCTGAACTCCTTAGATGATCCGGTAGTATCGTGT
AATTTACCAGTGGATGAAGTTAAAGCAAACCCATATCTATATCTAGCGGCCTCAGAT
TTAGGTGGTCATTTGGCTTATATTCAGTGGGATGGTGGATTCTGGTTTAGCGAAGCC
GTGTCTGCATACTTCATGGCATTTGAATCGGAACTGTCAAACGAACCTGCACAAAGT
GATTACGAGCCTCAGCTAAGTGAATACAGCTATAAAACTGGAATTTGA

EHT1 EcorI F: TTTGAATTCATGCCATCTTGGGGGTTCC

EHT1 NotI R: ATAAGAATGCGGCCGCAATTCCAGTTTTATAGCTG

Reaction system: 50 μL

5 X PCR buffer	10 μL
cDNA	2 μL
EHT1 Primer F	1 μL
EHT1 Primer R	1 μL
Primastar	0.5 μL
ddH_2O	35.5 μL

PCR reaction conditions:

98 ° C	2 min	
98 °C	10 S	
56 °C	15 S	35cycles
72 °C	1.5 min	

EHT1 gene knockout

1. Amplification the left and right recombinant arms

CCCAGGGCGCTCCAGCCCAGCCGACATTCACCAATTCAACGTTTCGTCACGATGATCAGAATATCTATA
TCAACGATACTGCCATTCCAAAAGAAGAGTTCATGTACGCTTTTGGGGGTACCTTAACCACTGGTCGTC
AGTCTGTGCATCGCAGCTTTGGCAACCCTGTACCCGCTGGACTTGCAGGATTCTCCTTAGGAACTTTTG
CCCTGGGTCTAATTCTTCTTCAGGCCAGAGGCGTAACGGACGTTAGCATTCTCATTGGAATGTTCCTTT
TTGTTTCAGGAGTAGTTGAAATAATTGCTGGTATTTGGTGTATCGTGGTGGAAAATACCTGGGCCAGTG
TAGTATTTCTGGGCTATGGTTCTTTCTGGCTTGGATATGCTGCAATTGTAACTCCGGCATTTGGTGTTGC
TGCTGCCTATGAAACAGCTGAAGATTTCAACAATGCCGTTGGAATGTACCTATGCCCTTGGGCAGTCTT
CAGTTTTATTCTCTGGAGTTGTACTTGGAAATCGACCTGGCCGCTCACCGCCTTAGTCTTCTGCATTTTT
ATGATATTTCTAGTATTGACAATTGGGTATTTTGTTAGCTCGGTGCCAACAATTAAAGCTGGAGGAGTCT
TTTGTTTCGTGGGTAGTCTCTTGGGATTTTACAACATGTATGCAGGTTTGGCTAATCCAACCAACAGTTA
TGTTGTTGTCCATCCTCTATTCATGCCTAATGCAGCCAAGCCAGCTAGTTAAGTAGTTTATTTACTTGAG
GAAATCCAACAACTTAGTTAACAGTTAACGTGTTTTTTTTTTTCTCGGACTCCTTATGGAGCCGCACCC
CCGGTGTGAAACCCCATCCCACATATGATCTCCCCCCGCACATGAAGGACTTAACAAGTCAGCCACCC
CCTCCTCCGCACCCTCAGTTATACGAAAATGGTAAGGAGGAATTACGTTTTCTCGGACTCCTAACACCC
CTGACACCCTATTTTAGATGTCCCTCTAAACCCCTCCTCAGCTTGCAGCACCCCATGATAGTTTTCAGTT
CATTTTCGTAGGTTAGTGACCATTTTCCTTGCAATATATATTGAAACGATTTCTTCCCTTAGCCATATTTC
ATTTCTCGTTTACCACCTACAGAGAGTTTTATATCAGCAGTTTGAAGTTTTGTTCCTTGTTCTAATGTCT
CAATCTCAATCAATTTCAGATCTTGCCCCAGATGTCGAAAAGGGCAACCCAATCGAACAAATACCAAC
CACATCAACTTTCCGTCACGATGATCAAAATATCTACATCAACGATACTGCTATCCCAAAAGAAGAGTT
CATGTACGCTTTTGGGGGTACATTGACTACTGGTCGTCAATCTGTGCATCGTAGTTTTGGTAATCCTGTC
CCTGCTGGTTTGGCGGCTTTTTCTTTGGGTACATTTTCACTAGGGCTTATCCTTCTGCAGGCCAGAGGT
GTGACAGATCCAACGATACTTGTGGGATTGTTTTTATTTGTTGCAGGGCTTGTGGAAATTATTGCAGGTA
TATGGTGCATTGTCGTGGAGAATACTTGGGCCAGTGTAGTTTTCTTGGGATACGGTTCTTTTTGGATAGC
TTATGGATGCATCGTGACCCCCGGATTGGGACTTGCTGCGGCCTACGATACAGTTGAAGACTTCAATAA
TGCTGTGGCAATGTTTTTATGTCCATGGACCGTACTGACTTTCATCCTTTGGAGTTGTACCTGGAAATCC
ACTTGGCCACTGACCGCCCTGGTTTTCGTGGTGTTCCTATTGTTCTTAGTACTGACGATTGGATACTACA
CCAGCTCAGCTGGTACTATCAAGGCTGGTGGAGCTGTATCGTTTGTTGGAAGTATACTTGGTTTTTACA
ATATGTATGCTGGAATTGCAGACTCTACAAACAGTTACTTGGTTGTCAAACCATTGTTTATGCCTCATGC
AGCAACTCCGAACTAGTATGAATGCACCAATCGTATAAAGTTTGACTCGACGTCACGATCTCTATTTTC
AATTAATCTGATTAAATGTATAACTCAAATTCCAGTTTTATAGCTGTATTCACTTAGCTGAGGCTCGTAAT

CACTTTGTGCAGGTTCGTTTGACAGTTCCGATTCAAATGCCATGAAGTATGCAGACACGGCTTCGCTAA
ACCAGAATCCACCATCCCACTGAATATAAGCCAAATGACCACCTAAATCTGAGGCCGCTAGATATAGAT
ATGGGTTTGCTTTAACTTCATCCACTGGTAAATTACACGATACTACCGGATCATCTAAGGAGTTCAGCA
GTACTGTTGGAGTGTAAATATTTACTAGCTTGGTTAAGGGAGATCCAGCTTGATAGTAATATTTTGGACT
TGCAAATCCATACGCTGGGGCAGTATATTTTCGATCAAAGTCGACGATTCTCCGTACCAAGTAATCTTT
GGACTTCTTTTCGTATGAGAATACTTCCGGTTTATCACGGTTTAGAATTGGGAAGTTAACTTTTACCAAA
TTTGACAAGAATGAAGCCACTGCAGGATCGAATAGATACTTTCCAGAGTAACTGTGGTTCAAATGATAA
TGGGAGTCCAGGAGGTCTACAGGTGCTCCGATAGTGACCGCACTTTTGATGAAGCTCTTCTTTCCTTCC
TCGCCAAGATAATTGAGAACCAGTGCAGACCCTAAAGAAAATCCCAAAAGATAGATAGGCTTGTTGGG
TATTTTCTTATGTAGCTCATCAATAAAGTAACGGAGATCATCTGTTTCGACGGCCGAGTAAAGTCTTCCT
GTTGACAATTTAGAGCGACAACATCCACGAGAGTTGAGTGTCACAACATTAAAACCAGCTTGATGAAG
ATGCTGGCTCACATCTCGTATTCCCGGTTCGTGCGAGCCACCGGCCAATCCGTGAATAATTACAACAAT
TGATCCAGAGTTTGAGGCCCAGTCAGCTTCCAGGTCTTGTTCTTCTTGCACTGTTAGGAATCTAGTCCG
CGGATGCAATCGAGGCCATCCTTCAGGTAAAGTCTGATTGTATAAATAATTCCATTCATCGATTTCTGTT
GGTGGATCAACTACAAAATCCGCAGTCGCTTGACCAAGATGTAAATGTGGATACTCATGATTTGCCTCA
GGATATCGAACCATGCATCTTCCATAGTATATTTGAAATTCTTTACTATAGTCAGCTTTGGACGCGTATAA
AGTCTGGAGAGCGCCATTGAACAGAAAAGGGTGAAGGTTGAATTTGTAGCCATCGCTGAGTTCAGGT
ACTAAAGTCTTAATAAATGTAGAAAAAGGAATGACATTCAAAGGTGCAAAGTCGTTCTTGCCAGACTT
CAGCTTGAGTGCCACCGATTTATCGCTGTAGGTAGCATGAATCTGATTATGGAACCCCCAAGATGGCAT
GTAGGTGTAATTTTTCGAAGAAAAGAAATCTAACGATTCGTCAATAAGGAAATGAAAAACGTTGTCCG
AACCGAACAGAATTGAACCTTTATTGCCGCAACCAATTTATTTATCTGAAAATGTAAATATTGAAATTTC
TACACGATTAACACTAATTATAGGTGTTCTCTCTAATGGTACTACACCTGGAGTGGTTGTTCACAATGAA
TTCCTCCATTGCTTAAGAAATGAATTGAGCCCATAGATGGTCCCTCCCACACCACAGGTTGGCATGATT
CTCATTTTCACATGCACAATTTTTTGTTGTGCAAACTAAGCTAACTTGACTCCATCCTTACACCATATTG
CCTTGAATGTTGGCGCCCCTATTGATTTTTGGTTTCACTTGCCATCTCATATAACCCTAGGACTTGAATAT
TCCTGTTCTAAACCCCCCCAAAGCTCCTAATGGCTTCTAAGAGGACCTCAGTTCGAGGGGAATCAGAT
TCAAATAACTATGCCTTATCAGACTCCTCCGATTTTGAAGACAATGGGCATCCGAGATTGTCAAAAAGA
CGATCTAGCCTGTTTTCTTTGAGTTCATTAAGCTCAGCCGAACCTTTACCACCTCCGGACGAAAAAGAT
TTTATTGCGTTTACATCTGACGACAGACATTTCTCTTTGCTTCGAAATCTTCATATGGCAGATTTCATAAC
GTTAATGAATGGTTTTTCCGGGTTTTATTCTATTGTTTCTTGTCTTCGTTTCACCTTGACTAAGCAAACTC
ACTACGTACAAAGGGCACACTTCTTCATAATTTTGGGTCTTTTTTTTGACTTTTTTGACGGTAAAGTTGC
CAGATTGCGCAACAAAGCATCATTGATTGGTCAGGAGTTGGACTCTTTAGCCGATCTGATTTCATTTGG
GGTTGCTCCCGCCTCTATTGCATTTTCCCTAGGTTTACAAACTACATTAGACGTCCTATGCCTAACATTT
CTGGTTCTGTGTGGACTCACCAGATTAGCTAGGTTCAACGTAACAGTGGCCAACATACCAAAAGATAT
CAAGGGTAAAGCTCACTATTTCGAAGGATTACCGATCCCTTCGACCTTGTGGCTAGTATTTAGTATGGC
ATTTTGTGTTTATAAAGGATGGTATGAGTATGAGGATGGCCTTCCTGGTCCATTATTATGGAAAGGAAAA
TTTTTTGAATTTCATTTGATTTCACTCTTTTTTGTCATTCAAGGATGTTTGATGACCTCCAAAAGTCTCA
AAATACCTAAACCTTGAATATGGATATGGTTGCATTATCAGAGTTCCGCCCATATTATATTCTGTGTAACC
AAATTTACAGGTAGATATAATCTCTTCTATTTACATATATTTACGAGCATGTCGCAATTTTTCATCGGAAG
GATTAACGAATTTCTTACTTTTCAATACCAAAAGCAGCAAAACTGTTGAAGCAAGAGACAACCCCCAC
CAGGTTACGAGATAATTTAAATGGTTGTTTGTGTAATCAACTTTTGGTACTTTGCCAATAGGTACTCCAG
CATTTACGAACTGTAATGGGCTGAATTCCAGCGATGAGTCTTGATAAGCAGAAACCTTTGCCTCCCCAA
CCATGGGAGCCCGAAATGTTTCCAGCTGTGATTGATCATCGTTGTTATCACTTTCACTTTGCCACCGCC

ACCTCCACAAGTTCCATAAACCTCCTGATGATTCATGCCCGATTTCGCTGGCCCTATCCTTAAGCCATTC
AGGATGATCCTTGAAATCTTGTACCACTTGGGCATATATTGGCTTGCAATTAGTCTCCTTGCACATTCGC
TCCAAATCGACATATCTGAACATATTTGTCCCTGGCTTATGTTCAATGTGAAACATTCCTTTCGATGGTG
GTACTTTCAAAAGACATTCTAAAGTTATTGTACCCTCTGGCATAGATAAGTTTTTTAGCCTTCTTTTTCC
TTGGTTCACTTTCCCTTCAGATATCCAGCCTCTTTCCACTAAAATTCTTCCACCTCCATTAGATCTTGTG
AAAGGAGTGACAAGGATATAGCCCTTTCTGTCATCGTGCAATCGGGGACCTACTAACAGTTCCTTTTCG
TAGTCAAATCTACCAGTTATGAGCACCTTGCGATACTCGAAGTTCTCGACATCCTCTTCCCTGAAGTTC
TTGGGAAGAGGAAGTGGCTTGTATGTCAATCTGTCTTCACACTCAGCTACTAGGGAATTTTTCCATTTC
AATCGCTGTAACTGCCAAAATCCGAGCCCCAAAGTTACTACTGGAGTTAGGCAAAGAAGGCCAAATAC
AACACTCCGCCCAGTGCGGTGCTTGTTTCCATCTCTCTTCAAGGATTTGTTAGATGTAATTGGTTTCCAA
TCGATATCTAAGGTCTTGGGTGTTGCAGGGGGTTTAGGTGGGTCGAAGTGCAGAAATCTGCGGATCAA
AGAGATACTAGTTCCATTTCTGAATTTTCGTACAAACATATGTCCTAACCAACACAAGAAGAAACTTCT
AGCTGTGTTTGAAAAGTGCTCGAATTTCAGGTTGATGGATGCCGAAGGGAAGATAAGTTACCTTTGCT
AAATTCCTTAGTGCTTGACGAGGAATTGAGACGATAGAAAGTAACAAGTTGAAACAAGTATAAAATCA
AGACAAGATAATATTAAACCAATTCATTGGGATTCACGTCCCATCTGTTTTATCAACTACCTGTCACCTC
TAAATTTCTCTCCACTGCGGGTTAGATCTTTACTCTAAATGCTATTAACTAAATC

The four primers below should be synthesized. Please note that there is a U in each primer.

EHT1O1	GGTCTTAAU TGGAAAATACCTGGGCCAGT
EHT1O2	GGCATTAAU CTGTCAAACGAACCTGCACA
EHT1A3	GGACTTAAU GTTGGCGCCCCTATTGATTT
EHT1A4	GGGTTTAAU CGGTGGCAAAGTGAAAGTGA

Running PCR on Genomic DNA to amplify recombinant region

PCR buffer 10X	1ul
dNTB 10mM	0.2 ul
Primer F 10uM	0.5 ul
Primer R 10uM	0.5 ul
Pol	0.2 ul
gDNA 10 ng tot	0.05 ul
(240 ng)	
H2O	7.55 ul
	----------
	10 ul

Ligation of the PCR products with the vector

Mix the following in a 0.2 ml PCR tube:

Tube 1 Tube 2

PCR product 1	75 ng/ul	5.3 ul
PCR product 2	~160ng/ul	2.5 ul
pH vector	315 ng/ul	0.6 ul

USER enzyme	1 ul
H2O	0.6 ul
Total	10 ul

Incubate at 37 C for 20 min followed by 25 C for 20 min.

Transformation of E. coli with the construct

Transfer the ligation reaction mix to a precooled 1.5 ml Eppendorf tube.

Add 100 ul of chemically competent E. coli DH5α cells (> $1*10^6$cfu) to the eppendorf tube.

Mix gently by tapping on the tube a couple of times and incubate for 30 min on ice.

Heat shock the cells by incubating them at 42 C for 45 sec, immediately afterwards return the cells to ice and incubate 2 min.

Add 900 ul of culture medium (SOC) preheated to 37 C, mix by inverting the tubes a couple of times and incubate for 1 hour at 37 C with shaking at 300 rpm.

Plate 100 ul directly on 1 plate LB + kanamycin.

Pellet the cells in a table top centrifuge for 1 min at 5000 rpm, discard about 9/10 of the supernatant and resuspend cells gently by pipetting up and down until cells have been separated. Plate approximately 50 ul on to single LB plate supplemented with 25 ug/ml kanamycin A.

Incubate plate overnight at 37 C.

Validating the presence of the inserts in E. coli transformants

Pick up ~20 E. coli colonies from the kanamycin plates using sterile pipet tip fitted on the pipet itself and suspend in 40 ul water.

PCR:

Stock		1 rect.
Buffer	10x	1 ul
dNTPs	10mM	0.2 ul
Primer F	10 uM	0.4 ul
Primer R	10 uM	0.4 ul
Taq*	---	0.1 ul
H2O	-	7 ul
Colony	-	1 ul

Each clone has two reactions
